# Necrotic Fibroid Mimicking Ovarian Torsion in a Second Trimester of Pregnancy

**DOI:** 10.7759/cureus.6172

**Published:** 2019-11-16

**Authors:** Neil P Larson, Rachel E Bridwell, Amber Cibrario, Jonathan Henderson

**Affiliations:** 1 Emergency Medicine, Brooke Army Medical Center, Fort Sam Houston, USA

**Keywords:** necrotic fibroid, acute abdomen

## Abstract

Uterine fibroids are incredibly common, benign smooth muscle tumors which range in severity of symptoms from asymptomatic to debilitating. While pain is frequently a symptom, degeneration and necrosis of uterine fibroids can rarely present as acute abdomen. The authors present the case of a pregnant female at 19 weeks' gestation, whose clinical and radiographic presentation mimicked that of ovarian torsion, ultimately requiring exploratory laparoscopy for definitive diagnosis.

## Introduction

Uterine fibroids, also referred to as leiomyomata, are benign smooth muscle masses of the uterus, with the prevalence rates exceeding 80% in some female populations [1]. While the majority of these masses may be asymptomatic, uterine fibroids can cause most commonly pelvic pain and dysfunctional uterine bleeding, and reproductive difficulties both conceiving and in 10%-40% of pregnancies [2,3]. Fibroid subtypes vary by anatomical location, broadly categorized into intramural, submucosal, and subserosal [2]. Arising from just the surface of the uterus, subserosal fibroids can also be pedunculated, or situated on a stalk. These two anatomical features generate the risk of fibroid torsion, which may present in an emergency department as acute abdominopelvic pain [2,3].

## Case presentation

**Figure 1:**
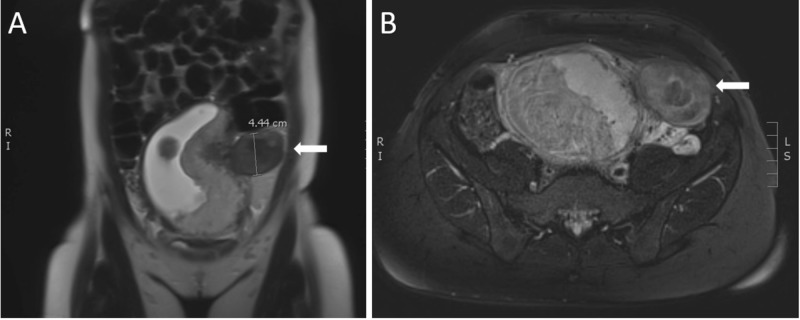
MRI revealing large left exophytic mass with internal hemorrhage (A) T1-weighted coronal image with height measurement depicting mass (white arrow). (B) T2-weighted axial image depicting the same mass (white arrow).

A 30-year-old primiparous female at 19 weeks' and 2 days' gestation by anatomical fetal ultrasound presented to the emergency department with a chief complaint of left lower quadrant abdominal and pelvic pain for the previous five days. The patient denied vaginal bleeding, discharge, or fluid leakage. Vital signs at triage were a blood pressure of 141/65 mmHg, a heart rate of 65 beats per minute, a respiratory rate of 16 respirations per minute, an oxygen saturation of 100% on room air, a temperature of 98.1 degrees Fahrenheit, and a pain level of 4/10. Physical examination was pertinent for a gravid uterus consistent with gestational dating and mild tenderness to palpation of the left lower quadrant of the abdomen without guarding or rigidity. Laboratory workup including complete blood count, basic metabolic panel, urinalysis, and quantitative hCG studies was unactionable. With concern for diagnosis of left ovarian torsion, a transabdominal ultrasound study was immediately performed, confirming a single, living intrauterine pregnancy with a fetal heart rate of 152 beats per minute. The study also revealed a left-sided, 5.8 x 4.0 x 5.8 cm exophytic fibroid at the left uterine fundus without internal vascular flow, replicated on an anatomical scan performed the day prior. While a pedunculated fibroid was listed as a possible diagnosis, no stalk was visualized on this lesion and the left ovary was also unable to be visualized, leading to the suspicion of ovarian torsion. Obstetrics and gynecology (OBGYN) was consulted for further evaluation and stated low current clinical suspicion for acute ovarian torsion at that time and arranged for next day clinic follow-up. Upon emergency physician re-evaluation of the patient, the patient appeared to be more tender than on previous assessment. An emergent pelvic magnetic resonance image (MRI) was ordered for continued suspicion of ovarian torsion. The MRI again revealed a left adnexal mass, with suggested internal hemorrhage and continued concern for ovarian torsion given no vascular flow present on sonogram (Figure 1A, 1B). With dynamic physical examination changes and pelvic MRI suggestive of ovarian torsion, the OBGYN team was reengaged. They re-evaluated the patient and took the patient emergently to the operating room for diagnostic laparoscopy. Surgical exploration revealed a large left-sided, necrosing, pedunculated fibroid with adhered omentum. Inspection of the bilateral ovaries and appendix was normal in appearance. Given the well-perfused, gravid uterus and large lesion size, the risk of bleeding outweighed the benefits of fibroid excision. The patient's abdomen was closed, managed with oral analgesia, and discharged the next day following uneventful postoperative recovery.

## Discussion

Ovarian torsion during pregnancy has an estimated incidence of five in 10,000 pregnant patients which is five times higher as compared to non-pregnant women, and may pose an immediate threat to the health of the mother, the pregnancy, and future fertility potential [4]. Similar to their non-pregnant counterparts, pregnant women with concern for torsion require immediate OBGYN consultation and surgical management for prompt detorsion. However, pain secondary to uterine fibroids may mimic the presentation of ovarian torsion or other sources of acute abdomen, and ultimately, in the patient above, diagnostic laparoscopy was required for definitive diagnosis.

Previous studies portray conflicting data regarding uterine fibroids changes during pregnancy, with data to support both increasing and decreasing size with hormone fluctuations [5]. However, a recent study demonstrated relatively consistent growth of uterine myomata in pregnancy with decreasing size of effect with progression from first to third trimesters [5]. While most uterine fibroids are asymptomatic in pregnancy, lesion size and location may contribute to pain. Acute abdominal pain affects up to 45% of patients with fibroids during pregnancy as compared to 18% in pregnant patients without fibroids [6]. Moreover, abdominal and pelvic pain was reported as the most common complication of fibroids in second and third trimesters of pregnancy with description of the syndrome of painful myomata of pregnancy [7]. This pain has been attributed to prostaglandin release due to lesion degeneration or progression to necrosis, a process occurring by fibroids outgrowing their blood supply [8]. Pain and necrosis may also rarely occur by torsion of pedunculated fibroids [3].

Diagnosis of uterine fibroids is often accomplished with ultrasonography, and absence of flow may be demonstrated in torqued or necrotic lesions, which is a rare complication. Similar to other ultrasonography, sensitivity can vary with operator dependence, and as in the case described, a pedunculated fibroid may be mistaken for an adnexal mass [9]. Pelvic MRI is then recommended for further evaluation, with an estimated 88%-93% diagnostic accuracy of identifying fibroid lesions [9].

Pelvic and abdominal pain due to fibroids in pregnancy is most often successfully treated conservatively with analgesia; however, surgical management in select cases may be pursued. While no unanimous consensus has been adopted, several guidelines for identifying surgical candidates have been proposed based on factors such as fibroid type, size, location, refractory symptoms, and proximity to the placenta [3,8].

In a retrospective case series, in which pregnant women underwent surgical myomectomy for recurrent pain, large or rapidly growing lesions, or myomata in the lower uterus or those affecting placental site, women gave birth to healthy weight babies with good APGAR scores, and without increased risk of preterm delivery, as delivery with previous myomectomy has demonstrated lower five-minute APGAR scores, increased rates of cesarean section, and increased rates of primary postpartum hemorrhage [8]. This demonstrated that while definitive surgical management is rare, it may be safe for both patient and fetus in carefully selected patients.

## Conclusions

Necrotic fibroid lesions are a rare source of acute abdominal pain in the emergency department and may mimic ovarian torsion in both patient presentation and radiographic evaluation. Emergent consultation with OBGYN is necessary for further evaluation including possible diagnostic laparoscopy for definitive diagnosis. With a formal diagnosis of uterine fibroids, analgesia is often sufficient for the treatment of symptoms. While surgery is generally avoided in pregnancy, carefully selected patients may safely benefit from myomectomy without adverse risks to the unborn child.
